# Shared decision-making about medication intake during lactation: A prospective longitudinal study in Greece

**DOI:** 10.18332/ejm/149830

**Published:** 2022-08-01

**Authors:** Maria Tigka, Dimitra Metallinou, Lemonia Pardali, Katerina Lykeridou

**Affiliations:** 1 Department of Midwifery, School of Health and Care Sciences, University of West Attica, Athens, Greece; 2 Obstetric Emergency Department, General and Maternity Hospital ‘Helena Venizelou’, Athens, Greece; 3 Neonatal Department, ‘Alexandra’ General Hospital, Athens, Greece

**Keywords:** shared decision-making, medication intake, healthcare professionals, lactating women, lactation, breastfeeding

## Abstract

**INTRODUCTION:**

The need for medication intake during lactation may affect women’s decision on breastfeeding initiation, duration or cessation. We investigated shared decision-making about medication intake (MI) during lactation by breastfeeding women.

**METHODS:**

The study was conducted in five maternity hospitals in Greece (January–August 2020). A total of 283 mothers participated in the study. Data were obtained through a self-designed questionnaire. Mothers answered the questionnaire before discharge and were followed up by phone interviewing at one, three and six months postpartum. Information about breastfeeding status, reasons for cessation and MI during lactation were gathered.

**RESULTS:**

In total, 30.7% of the mothers were receiving medication due to a pre-pregnancy chronic condition but only 23.7% maintained it during lactation; 48.4% of mothers stated that they would avoid MI during lactation as a personal attitude and 45.2% were satisfied with the information provided by healthcare professionals (HPs) concerning MI during lactation. But, 66.1% of the mothers indicated the necessity of further guidance. Mothers with higher education, Greek ethnicity and vaginal delivery participated significantly in the decision-making process concerning MI during lactation (p=0.001, p=0.001 and p=0.01, respectively). Mothers who shared decision-making, primarily consulted a pediatrician (p=0.02) and were more likely to maintain full and mixed breastfeeding for one month postpartum, rather than cease breastfeeding (p=0.005). Breastfeeding duration of all indicators was for a mean of 110 days (SD: 74.58).

**CONCLUSIONS:**

Advancing HPs’ evidence-based knowledge, communication skills, confidence and competence in breastfeeding management will contribute favorably to breastfeeding indicators and maternal satisfaction regarding professional counseling.

## INTRODUCTION

Shared decision-making is a two-way communication process between a patient and one or more healthcare professionals (HPs) through which patients understand the nature of their disease, based on facts or information. Considering the benefits, risks, limitations and alternatives of a medical procedure, either diagnostic or therapeutic, patients are empowered afterwards to consent or refuse the offer of certain provided healthcare or select among the given approaches^1^.

Concerning breastfeeding, all mothers should be informed and supported throughout the perinatal period by specialized HPs. Partners, close relatives and social environment as well, are of primary importance in supporting and encouraging women to initiate breastfeeding as soon as possible after birth^2^. Physicians, midwives, nurses and lactation consultants have been associated with significant positive influence on breastfeeding initiation rates and duration when they provide a well-structured educational breastfeeding program to mothers^3^. However, much remains to be done to make exclusive breastfeeding (EBF) the norm for infant feeding, as according to Centers for Disease Control and Prevention (CDC) for the year 2018, a large percentage of mothers (83.9%) started breastfeeding but only the 25.8% maintained EBF for a 6-month period^4^. Further demonstration of breastfeeding global trends has been recently presented in a study by Neves et al.^5^ who analyzed, from 2000 to 2019, seven feeding indicators among children younger than 2 years, including data from 113 countries around the world. The results revealed that upper middle-income countries are struggling to reach the World Health Assembly’s initial target of 50% by 2025, as the reported EBF rate for infants younger than 6 months was 37% in 2019. In contrast, low-income and middle-income countries are already close to the international goal with an approximate EBF rate of 49% in the same year.

Multiple other factors additionally have an impact on breastfeeding initiation and duration, including maternal education level, age and pre-pregnancy body mass index^6^, smoking habits, psychological status and introduction of a pacifier^7^. The need of medication intake, however, usually plays a crucial role in breastfeeding cessation. A fairly large percentage of mothers (>50%) during lactation receive medication such as oral contraceptives, systemic antibacterials/antibiotics, nonsteroidal anti-inflammatory drugs, dermatological agents, vitamins, iron preparations, analgesics/antipyretics or drugs for various chronic diseases^8,9^.

Bio-availability, molecular weight, ionization properties, protein binding and lipid solubility should be taken into consideration when a medicine is prescribed to a lactating woman, as these factors will determine the amount of excreta into the breast milk. Most medicines, especially drugs, excrete in breast milk to some extent, but the effects on the breastfed child depend on its age as well as on the concentration, half-life, toxicity and daily dose of the substance^10^. Probable adverse side effects on children are vomiting, diarrhea, lethargy, poor weight gain, hypothyroidism, hyperactivity, urticaria and hepatotoxicity. Thus, medication should be used in low dosages most of the time and for as short a time as possible^11^.

The HPs should be regularly updated on the categories of medication that are compatible with breastfeeding or contraindicated, as lactating mothers are remarkably concerned about the safety and potential effects of pharmaceutical substances on milk production and their children and, thus, often turn their attention to complementary and alternative medicines (CAMs) or methods^12,13^. However, evidence for CAMs (generally defined as any type of product manufactured from plants or with natural origin) in comparison with conventional ones, is very lacking^14^. Decision-making regarding medication intake during lactation is based on HPs’ latest knowledge and skills in transmitting scientifically valid information concerning the benefits of breastfeeding and the risk of drug exposure^15^. Lactating women should be educated about the reliable sources of information with regard to medication during lactation and encouraged to be actively involved in decision-making with their healthcare provider^16^.

Low breastfeeding rates and breastfeeding cessation are often attributed to HPs’ insufficient evidence-based knowledge and advice regarding pharmaceuticals throughout lactation^8,17^. Indeed, the provision of care to a lactating woman is a major challenge that HPs should focus on in the future.

The aim of the present study was to investigate shared decision-making about medication intake during lactation by breastfeeding women in Greece. Objectives of the study were to assess lactating women’s: 1) attitude towards reception of medicines, 2) degree of satisfaction as regards the information provided by HPs about medications; and 3) involvement in decision-making regarding medication intake. In the present study ‘medication intake’ refers to prescribed and over-the-counter (OTC) medication.

## METHODS

### Study design

This is a prospective descriptive observational longitudinal study, conducted during the period January–August 2020. The study was approved by the Ethics Committees of five maternity hospitals, three public and two private ones, in Attica, Greece. All are tertiary and the three public are referral hospitals for maternity services to women belonging to the prefecture of Attica and the provinces.

### Sample and setting

The final sample size was initially determined by specific time and condition constraints as it was mandatory to complete the recruitment within one month in each maternity hospital and include as many mothers as possible. This study was conducted during the COVID-19 pandemic so its impact on sampling recruitment methods should not be ignored.

According to the inclusion criteria mothers should: a) communicate effectively in the Greek language, and b) possess a telephone number so that the follow-up conversation would be feasible in the future. A total of 350 mothers were primarily approached and invited to take part in the study. Of these, 325 consented in participating and filling in the questionnaire of the subsequent study (response rate 92.8%). Finally, 283 mothers were enrolled, as 42 were excluded from the study. Exclusion criteria were: a) incomplete answers/loss of the questionnaire within hospitalization (n=17), b) lack of response in one out of three time points of the follow-up (n=20), and c) mothers whose infant was diagnosed with a life-limiting condition or poor prognosis (n=5).

### Measurements

Data collection was performed by using a questionnaire designed by the first author after a thorough relevant literature review^13,18,19^.

Initially, the questionnaire was distributed to five experts to evaluate its content and check the clarity of the questions. Subsequently, it was tested in a pilot study, during December 2019, with a sample of 50 mothers in order to identify potential problem areas and deficiencies in the research instrument and protocol, prior to the implementation in the full study (the pilot study’s participants were not included in the present study). The final form of the questionnaire included both open-ended and closed questions.

The first part of the questionnaire concerned demographic, socioeconomic and lifestyle characteristics (maternal age, ethnicity, marital status, employment status, education level, type of hospital, and cigarette consumption). The second part of the questionnaire examined the medical, maternity and lactation history (parity, maternal body weight prior to pregnancy and birth, duration of gestation, mode of delivery, newborn’s sex and birth weight, breastfeeding duration of a previous child, 6-month breastfeeding follow-up and reason for breastfeeding cessation) as well as relevant data about medication intake. Finally, the third part of the questionnaire included information about the lactating women’s: 1) attitude towards reception of medicines, 2) degree of satisfaction as regards the information provided by HPs about medications, and 3) involvement in decision-making regarding medication intake. Follow-up was based on telephone communication at the 1st, 3rd and 6th month postpartum. Definitions of full breastfeeding (FBF), mixed breastfeeding (MBF) and any breastfeeding (ABF), which results from FBF and MBF, followed the WHO classification^20^.

### Data collection

The first author informed the eligible mothers of the purpose and the nature of the study 24 hours after childbirth and notified them that the questionnaire should be answered before being discharged from the maternity hospital (the average nationwide in-patient stay in the postpartum ward is four days). In this way, sufficient time was given to all prospective participants to consider whether or not they are interested in proceeding to the process of consent. Recruitment was consistent with all ethical considerations being acknowledged and there were no direct personal benefits of participation in this study.

Once mothers voluntarily agreed to participate, they were given an envelope containing the questionnaire of the study and an informed consent form. The questionnaire was distributed in person by the first author in order to: 1) avoid systematic errors due to postal handling, 2) provide explanations to the participants when necessary, and 3) achieve clarity of the answers.

Upon completion the questionnaire and the signed consent form were returned to the first author in a closed envelope, in order to maintain the anonymity and confidentiality of the data. One, three and six months after the discharge from the maternity hospital, mothers were followed up by phone interview to retrieve information about medication intake. Moreover, breastfeeding status and the reasons for breastfeeding cessation were investigated. The response rate of the follow-up was 93%. Coding of all participants was automatically created by the database used to preserve de-identification.

### Statistical analysis

Continuous variables are presented as means with standard deviation, and categorical variables as frequencies. Baseline characteristics were compared by applying a chi-squared test (χ^2^) for categorical variables, and independent samples t-test for continuous variables. Data analysis was performed using SPSS version 20. A probability level of less or equal to 0.05 was considered significant.

## RESULTS

Maternal baseline sociodemographic characteristics including age, education level, family status and number of family members, employment before pregnancy, type of maternity hospital where the birth occurred, region of residence and ethnicity are presented in Table 1. The mean age of the studied sample was 33.27 ± 5.11 years. The majority of mothers were married, Greek, and highly educated. When asked about their employment status before pregnancy, most (77.4%) answered that they were professionally active. Over half of the mothers surveyed (77.1%) reported that they were living in the region of Attica.

**Table 1:** Maternal baseline sociodemographic characteristics, Attica/Greece, 2020 (N=283)

*Baseline characteristics*	*n (%)*
**Education level**	
Primary to high school	63 (22.2)
University	186 (65.7)
Postgraduate studies	34 (12.1)
**Employment before pregnancy**	
Yes	219 (77.4)
No	64 (22.6)
**Type of maternity hospital**	
Private	135 (47.7)
Public	148 (52.3)
**Region of residence**	
Attica	218 (77.1)
Provinces	65 (22.9)
**Ethnicity**	
Greek	255 (90.1)
Other	28 (9.9)
**Family status**	
Married	265 (93.6)
Unmarried	18 (6.4)
	***Mean ± SD***
**Number of family members**	3.63 ± 0.85
**Age** (years)	33.27 ± 5.11

Maternal history related to pregnancy, birth and lactation is presented in Table 2. Remarkably, well over half (69.6%) of the mothers questioned had given birth via caesarean section. The majority of mothers were willing to breastfeed (91.5%) and some had a previous breastfeeding experience (45.2%). Within the first 24 hours after birth, a large percentage of mothers (47%) were fully breastfeeding their infants. At 1 month and 3 months, a similar percentage continued with FBF (49.8% and 45.6%, respectively), while at 6 months after delivery the percentage declined to 9.5%. With regard to MBF, 44.5% of mothers maintained MBF at 24 hours after birth, which dropped to 30.1% and 15.2% at 1 and 3 months postpartum, respectively, rising again to 38.2% at 6 months postpartum. The rate of ABF at 24 hours after birth was 91.5%, which dropped to 79.9%, 60.8% and 47.7% at 1, 3 and 6 months postpartum, respectively. The breastfeeding mean duration of all breastfeeding indicators was 110.95 ± 74.58 days. Over the months, more and more mothers ceased breastfeeding with the rate eventually reaching 52.3% (148/283) at 6 months postpartum. The most frequent reasons for breastfeeding cessation were the perceived low milk production (42.6%; 63/148) and medication intake (12.2%; 18/148). In regard to cigarette consumption, few mothers (8.1%; 23/283) continued smoking during lactation, if we take into consideration that 32.9% of the mothers were smokers prior to pregnancy.

**Table 2:** Maternal history related to pregnancy, birth and lactation, Attica/Greece 2020 (N=283)

*Variables*	*n (%)*
**Sex of the newborn**	
Female	123 (43.5)
Male	160 (56.5)
**Mode of delivery**	
Vaginal delivery	86 (30.4)
Caesarean section	197 (69.6)
**Gravida**	
Primigravida	138 (48.8)
Multigravida	145 (51.2)
**Analgesia during labor**	
Epidural	260 (91.9)
Local anesthesia	10 (3.5)
General anesthesia	13 (4.6)
**Previous breastfeeding experience**	
Yes	128 (45.2)
No	155 (54.8)
**Willing to breastfeed**	
Yes	259 (91.5)
No	24 (8.5)
**Breastfeeding status 24 hours after birth**	
Full	133 (47)
Mixed	126 (44.5)
Any	259 (91.5)
Breastfeeding cessation	24 (8.5)
**Breastfeeding status one month after birth**	
Full	141 (49.8)
Mixed	85 (30.1)
Any	226 (79.9)
Breastfeeding cessation	57 (20.1)
**Breastfeeding status three months after birth**	
Full	129 (45.6)
Mixed	43 (15.2)
Any	172 (60.8)
Breastfeeding cessation	111 (39.2)
**Breastfeeding status six months after birth**	
Full	27 (9.5)
Mixed	108 (38.2)
Any	135 (47.7)
Breastfeeding cessation	148 (52.3)
**Breastfeeding cessation until 6th month**	
Yes	148 (52.3)
No	135 (47.7)
**Reason for breastfeeding cessation**	
Perceived low milk quantity	63 (42.6)
Breastfeeding attachment problems	10 (6.8)
Maternal choice	13 (8.8)
Nicotine consumption	4 (2.7)
Employment conditions	6 (4.0)
Nipple trauma	4 (2.7)
Mastitis	7 (4.7)
Maternal psychological issues	5 (3.4)
Flat nipples	4 (2.7)
Previous negative breastfeeding experience	4 (2.7)
Medication intake	18 (12.2)
Other	10 (6.8)
**Smoking before pregnancy**	
Yes	93 (32.9)
No	190 (67.1)
**Smoking during lactation**	
Yes	23 (8.1)
No	260 (91.9)
	***Mean ± SD***
**Number of newborns**	1.61 ± 0.75
**Duration of pregnancy** (weeks)	38.30 ± 1.53
**Newborn’s birth weight** (g)	3.132 ± 473.80
**Mother’s weight before pregnancy** (kg)	66.64 ± 14.6
**Mother’s weight before birth** (kg)	79.62 ± 14.0
**Breastfeeding duration** (days)	110.95 ± 74.58
**Breastfeeding duration of previous breastfeeding experience** (days)	288.63 ± 296.52

Approximately one-third of the mothers (30.7%; n=87) were receiving medication due to a pre-pregnancy chronic disease but only 23.7% maintained it during lactation (Table 3). Such conditions included endocrine disorders (diabetes, hypothyroidism, hyperthyroidism, polycystic ovary syndrome; n=51), autoimmune diseases (ankylosing spondylitis, Hashimoto disease, psoriasis, autoimmune gastritis, Crohn’s disease; n=14), diseases of the hematopoietic system (anemia, thrombophilia; n=8), mental health disorders (generalized anxiety disorder, depression; n=5), neurological disorders (epilepsy, carpal tunnel syndrome, migraine; n=4), respiratory diseases or disorders (chronic bronchitis, asthma; n=3), skeletal disorders (disk herniation; n=1), and genitourinary disorders (chronic pyelonephritis; n=1).

**Table 3:** Information related to medication intake during lactation, Attica/Greece 2020 (N=283)

*Variables*	*n (%)*
**Medication intake due to chronic disease before pregnancy**	
Yes	87 (30.7)
No	196 (69.3)
**Medication intake due to a chronic disease during pregnancy**	
No	211 (74.5)
Same dosage and frequency as before pregnancy	21 (7.4)
Same dosage with reduced frequency	3 (1.1)
Same dosage with increased frequency	42 (14.9)
Change of the medicinal product	6 (2.1)
**Medication intake due to chronic disease during lactation**	
No	216 (76.3)
Same dosage and frequency as before pregnancy	18 (6.4)
Same dosage with reduced frequency	3 (1.1)
Same dosage with increased frequency	41 (14.5)
Change of the medicinal product	5 (1.7)
**Attitude towards medication intake**	
Positive to medication intake	30 (10.6)
Avoid medication intake	161 (56.9)
Never take medications	92 (32.5)
**Possible reasons for refusing medication intake during lactation after hospitalization**	
Fear of harming the newborn	88 (31.1)
Avoiding taking medications is my ‘personal attitude’	137 (48.4)
If necessary, I will take medications	58 (20.5)
**Maternal opinion towards medication intake during lactation**	
Breastfeeding women can take more medications than pregnant women	34 (12.0)
Breastfeeding women can take the same medications as pregnant women	99 (35.0)
Breastfeeding women can take less medications than pregnant women	150 (53.0)
**Maternal satisfaction with information provided from healthcare professionals concerning medication intake during lactation**	
Yes	128 (45.2)
No	155 (54.8)
**Maternal need for more information concerning medication intake during lactation**	
Yes	187 (66.1)
No	96 (33.9)
**Clarified information from healthcare professionals concerning medication intake during lactation**	
Yes	130 (45.9)
No	153 (54.1)
**Maternal involvement in decision-making concerning medication intake during lactation**	
Yes	201 (71.1)
No	82 (28.9)
**Source of information concerning medication intake during lactation**	
Obstetrician	111 (39.2)
Midwife	23 (8.1)
Pediatrician	123 (43.5)
Pharmacist	3 (1.1)
Scientific/professional websites/forums	18 (6.4)
Breastfeeding counseling in the hospital setting	2 (0.7)
Certified lactation consultant	3 (1.0)
**Maternal reaction after unclear counseling concerning medication intake during lactation**	
Continue breastfeeding, without medication intake	71 (25.1)
Cease breastfeeding, start medication intake	5 (1.8)
Continue breastfeeding, start herbal products	3 (1.1)
Seek information from other sources	204 (72.0)

Almost half of the mothers (48.4%) stated that they would avoid medication intake during lactation as it was their ‘personal attitude’. Moreover, 53% of the participants erroneously considered that during lactation women can take less medications than during the period of pregnancy^21^.

About half of the studied sample (45.2%) was satisfied with the information provided by HPs concerning medication intake during lactation, but 66.1% of the mothers indicated the necessity of further guidance. Most (43.5%) of the mothers surveyed obtained information concerning medication intake during lactation from a pediatrician, in comparison with other HPs and scientific/professional websites or forums. When mothers were not satisfied with the counseling provided, most sought guidance from other sources (72%) and some (25.1%) eventually resumed breastfeeding without medication intake (Table 3).

Our results highlight that mothers’ involvement in decision-making concerning medication intake during lactation is significantly correlated with sociodemographic and clinical factors, such as education level, ethnicity, mode of delivery, source of information, breastfeeding status one month after birth, and maternal reaction after unclear counseling (Tables 4 and 5). More specifically, mothers with higher levels of education and Greek ethnicity as well as mothers who gave birth vaginally, participated significantly in the decision-making process concerning medication intake during lactation (p=0.001, p=0.001 and p=0.01, respectively). Moreover, the analysis revealed that mothers who shared decision-making concerning medication intake during lactation: a) were more likely to maintain FBF and MBF at one month after birth than stop breastfeeding (p=0.005), and b) primarily received information from a pediatrician (p=0.02). Interestingly enough, mothers who were involved in decision-making and believed that counseling concerning medication intake during lactation was unclear, searched additional information from other sources or obtained a herbal product that was presumed by them to be safer for breastfeeding (p=0.01). Other contributing factors, including maternal age, number of children, number of family members and duration of pregnancy were not associated significantly with maternal involvement in decision-making as regards medication intake during lactation (results not shown in Table 4).

**Table 4:** Sociodemographic factors related to maternal involvement in decision-making concerning medication intake during lactation, Attica/Greece 2020 (N=283)

*Maternalt involvement*	*Sociodemographic factors*			*p*
**Education level,** %				
	Primary to high school	University	Postgraduate studies	
Yes	60.3	71.4	91.2	0.001*
No	39.7	28.6	8.8	
**Employment before pregnancy,** %				
	Yes	No		
Yes	71.2	71.4		0.97
No	28.8	28.6		
**Type of maternity hospital,** %				
	Private	Public		
Yes	47.3	52.7		0.74
No	49.4	50.6		
**Region of residence,** %				
	Attica	Province		
Yes	77.1	22.9		0.80
No	76.5	23.5		
**Ethnicity,** %				
	Greek	Other		
Yes	74.1	44.4		0.001*
No	25.9	55.6		
**Family status,** %				
	Unmarried	Married		
Yes	72.2	71.2		0.92
No	27.8	28.8		

* Significant values.

**Table 5:** Clinical factors related to maternal involvement in decision-making concerning medication intake during lactation, Attica/Greece 2020 (N=283)

*Maternal involvement*	*Clinical factors*							*p*
	**Mode of delivery,** %							
				Vaginal delivery	Caesarean section			
Yes				34.5	65.5			0.01*
No				19.8	80.2			
	**Analgesia during delivery,** %							
			Epidural	Local	General			
Yes			90.5	4.0	5.5			0.44
No			95.1	2.5	2.5			
	**Breastfeeding status one month after birth,** %							
			Full breastfeeding	Mixed breastfeeding	Breastfeeding cessation			
Yes			51.7	32.8	15.4			0.005*
No			45.7	22.2	32.1			
	**Medication intake during lactation,** %							
		None	Same dosage and frequency as before pregnancy	Same dosage with reduced frequency	Same dosage with increased frequency	Change of the medicinal product		
Yes		75.6	5.5	1.0	13.9	2.5		0.28
No		69.1	8.6	1.2	16.0	0		
	**Personal attitude towards medication intake,** %							
			Positive to medication intake	Avoid medication intake	Never take medications			
Yes			11.4	54.7	33.8			0.44
No			8.6	63.0	28.4			
	**Maternal satisfaction with information provided from healthcare professionals concerning medication intake during lactation,** %							
				Yes	No			
Yes				48.3	51.7			0.12
No				38.3	61.7			
	**Clarified information from healthcare professionals concerning medication intake during lactation,** %							
				Yes	No			
Yes				49.2	50.8			0.17
No				38.3	61.7			
	**Maternal reaction after unclear counseling concerning medication intake during lactation, %**							
			Continue breastfeeding, without medication intake	Cease breastfeeding, start medication intake	Continue breastfeeding, start herbal products	Seek information from other sources		
Yes			20.9	1.0	1.5	76.6		0.01*
No			35.8	3.7	0	60.5		
	**Source of information concerning medication intake during lactation, %**							
	Obstetrician/gynecologist	Midwife	Pediatrician	Pharmacist	Scientific/professional websites/forums	Breastfeeding counseling in the hospital setting	Certified lactation consultant	
Yes	33.8	9.5	46.3	1	8.5	0.5	0	0.02*
No	53.1	4.9	37.0	1.2	1.2	1.2	1.2	

* Significant values.

Finally, no significant correlations were observed between: a) maternal satisfaction with the information provided from HPs concerning medication intake during lactation and breastfeeding status or cessation; and b) maternal attitude towards medication intake and ethnicity, family status or education level (results not shown).

## DISCUSSION

To our knowledge, the present study is the first to identify sociodemographic, cultural and individual factors that affect women’s decision-making about medication intake during lactation in the Greek setting.

Lactation is a very sensitive period during which women are searching for information regarding the risks and benefits of medications so as to use them with safety for themselves and their child^22^. Previous studies report that mothers who were in need of medication intake during lactation stopped breastfeeding earlier than desired^17^. Many women may confuse medication intake as a barrier to breastfeeding, therefore leading to a decline in breastfeeding rates. Lack of adequate and appropriate information provided by HPs contributes to early cessation of breastfeeding or insufficient healthcare for breastfeeding women^23,24^.

In our study, the FBF rate at 24 hours after birth reached 47%, a finding that is in line with the 51.1% of the latest National Breastfeeding Study conducted by the Institute of Child Health (ICH) during the year 2017 in Greece^25^. Besides, the FBF rate at six months after birth was found to be 9.5% in the present study compared to 0.78% of the aforementioned National Study. Additionally, ABF rates of the current study at 24 hours, and at one, three and six months after birth (91.5%, 79.9%, 60.8% and 47.7%, respectively) approached the respective ones of the National Study (90.8%, 79.8%, 63.4% and 45.3%, respectively). Thus, the results of our study are consistent with the latest National Study, with the only exception of FBF rates that were found to be higher in our study at six months postpartum. In our opinion, there are three possible explanations for this observation. Firstly, two out of the five participating hospitals are certified as ‘breastfeeding – friendly hospitals’ and enhancements in hospital practices may have contributed in favor of breastfeeding rates. Secondly, there is an ongoing Greek program called ‘Alkyoni’, which is a national initiative implemented by the ICH with the aim to protect, promote and support breastfeeding through awareness campaigns, educational activities for healthcare professionals and parents, and a breastfeeding phone helpline. The aforementioned actions of this program could well be responsible for the increase in breastfeeding rates occurring during the last years in Greece. Furthermore, it cannot be ruled out that the COVID-19 pandemic had a beneficial effect on breastfeeding rates in the present study due to lockdowns, home confinement and teleworking^26^. Lastly, in the present study, the most frequent reason given for breastfeeding cessation within the first six months postpartum, was the perceived low milk quantity, a finding that confirms previous results^27,28^.

Although most mothers (48.4%) in our study reported that they would possibly refuse medication intake during lactation after hospitalization as a personal attitude, eventually it was the second most important cause of breastfeeding cessation, recorded by a small but nevertheless, not negligible percentage of mothers (12.2%). That result differs from the data obtained in earlier Greek studies^7^, where fatigue, ablactation and general breastfeeding problems were more usual reasons for discontinuation than medication consumption.

Pediatricians and obstetricians were reported to play a significant role in the maternal decision-making process concerning medication intake during lactation in the current study. That finding was not surprising, as non-Greek participants were few (9.9%) and in Greek culture mothers have a strong personal interaction with these HPs, especially due to the fact that they experience consultation and continuing support during the postpartum period in their private offices. Besides, a high percentage of mothers (47.7%) had given birth in a private maternity hospital where the provided services encourage the close interrelationship between pediatricians/obstetricians and mothers as well. Similarly, Al-Sawalha et al.^16^ in a national study, assessed the beliefs and attitudes of 903 Jordanian women towards medication intake during lactation and concluded that the majority of them consulted their physician or pharmacist, so as to initiate or alter any medication. Contrary to expectations, midwives in both studies were not found to be a key-person in this process although their promoting and supporting role in breastfeeding has been previously acknowledged^29^.

These data highlight the need for comprehensive breastfeeding educational programs and communication skill courses to be incorporated in midwifery curricula so as to address the former disparity. Nonetheless, there is evidence that many mothers, especially those with higher education level, integrate information relating to medications during lactation from multiple resources so as to decide whether or not they will proceed with their consumption^11,22^.

A recent study in France, aimed to identify HPs’ perceptions of breastfeeding women’s attitudes and behaviors about medication use^24^. The results revealed that mothers’ refusal to receive conventional medications during lactation was a burning issue among HPs as according to them, mothers characterized medications inessential and preferred enduring pain and discomfort during breastfeeding rather than receiving some. Besides, HPs reported that mothers took medication only when it was absolutely necessary. These observations corroborate with our data, as women in our study recorded that they would avoid medication intake during lactation (48.4%) as a personal attitude or take medicines only when necessary (20.5%). It cannot be neglected that HPs’ role to offer well-informed counseling is of utmost importance and should be properly organized by all responsible health carers^30,31^. Healthcare professionals are obliged to thoroughly and adequately inform breastfeeding women about the safety of medications and their possible side effects, and to provide alternative options in circumstances where medications cannot be used^32^.

Furthermore, non-involvement in decision-making about medication intake during lactation was significantly correlated with non-Greek ethnicity. It is probable that foreigners are: 1) convinced with the guidance Greek HPs’ provide, 2) lacking access to other resources concerning a cross-check of the given information, and 3) disempowered to express their opinion on medication intake. Given that our finding is based on a limited number of non-Greek mothers (9.9%), the results from such analysis should consequently be treated with considerable caution.

Following on, the level of education, mode of delivery and breastfeeding status one month after birth, seemed to be other important background variables for shared decision-making about medication intake during lactation. It may be assumed that women in our sample were involved more due to their high education level and great access to information and healthcare. Delivering vaginally and maintaining FBF and MBF at one month after birth point to the likelihood that most women had attended prenatal educational programs and/or received considerable support and assistance by HPs during birth and postpartum period.

Finally, we feel strongly that the importance of our study lies in the fact that maternal characteristics of the study sample, when compared with data from the Greek Statistical Authority (GSA), were found to reflect the female population of Greece to a significant extent. More specifically, in the present study, 93.6% of the women involved were married, 90.1% had Greek ethnicity and the mean age was 33.27 ± 5.11 years. The respective data of the GSA revealed that 90.8% of the women who gave birth during 2020 were married, 85.2% were Greek and the mean age was 31.6 years^33^. As for the mode of delivery, the national rate of caesarean sections in Greece has increased the last years, reaching 56.8% in 2016 according to the WHO report^34^, whereas, in our study, disappointingly, a much higher percentage of women had delivered via caesarean section (69.6%). We hypothesize that the increased rate could be a result of the COVID-19 pandemic, as the present study took place during that period. Arab et al.^35^ has stated that delivering via the caesarean route is becoming common, both in the infected and non-infected pregnant population.

### Strengths and limitations

This study has gone some way towards enhancing our understanding of mothers’ decision-making about medication intake during lactation, especially in the Greek setting. We are aware though, that our research had some strengths and limitations. Even though not cost-effective, the fact that the questionnaire was distributed in person by the first author who was available for explanations and clarifications, increases the reliability and therefore the strength of our survey. Additional strengths were the high response rates both during hospitalization and follow-up, as well as that participants came from five maternity hospitals, either private or public, indicating a more representative sample.

A limitation of our study is the small sample size which possibly influenced the results obtained, mostly referring to the lack of significant correlations either between maternal satisfaction with the information provided from HPs concerning medication intake during lactation and breastfeeding status/cessation or between sociodemographic characteristics and maternal attitude towards medication intake. Nevertheless, maternal involvement in decision-making concerning medication intake during lactation was associated significantly with sociodemographic and clinical characteristics, such as education level, ethnicity, mode of delivery, and breastfeeding status one month after birth. Another limitation was that the sample was drawn from a single big city although the hospitals included serve women from the provinces too. In any case, the generalization of the findings is restricted to the national level. Finally, guidelines and counseling among maternity hospitals and HPs might differ and the quality of information provided could not be assessed.

### Recommendations for clinical practice and future research

Healthcare professionals and mothers must make decisions for medication intake during lactation integrating the obtainable data on the maternal and neonatal/infant impact of disease. Such data should include clinical pharmacokinetics, maternal and neonatal/infant safety, evidence-based sources of information, and available guidelines from scientific committees^36^.

Initially, healthcare professionals should offer a thorough risk-benefit analysis before prescribing or advising medication during lactation. Mothers should also be aware of all the dispensable drugs and OTC medications before considering a pharmaceutical product, if numerous for a disease, along with their adverse side effects. Resources for information, both for HPs and mothers, on drug safety and dosing in lactation, must be universal, easily accessible and frequently updated. Indeed, databases on drugs and lactation provide evidence-based medicine information but ideally, the drug label should include a narrative section related to lactation in any case. The ultimate goal is mothers to be offered continuous postpartum care from HPs who will regularly reassess the medications taken during lactation.

Furthermore, continuing educational courses which integrate the shared decision-making approach in daily clinical practice should be mandatory for HPs. Such courses are likely to introduce the concept of shared decision-making with a scientific approach and can potentially improve HPs’ communication skills, enhance mother’s medication compliance and increase mothers’ satisfaction with the provided care.

Development of effective animal models and new research techniques that evaluate and assess the use and safety of medications during lactation may close research gaps. Future research should focus on long-term effects on premature/term neonates and infants who were exposed to medicines during lactation. Studies expanding the follow-up period beyond 6 months are needed so as to address potential linkage between neurodevelopmental disorders and exposure in medicines during lactation. Additionally, future studies on shared decision-making about medication intake during lactation are recommended in order to clarify the associated influencing factors. Further work is required on larger sample sizes that will be guided by uniformly accepted sources of information and will be well-defined in terms of ethnicity, culture, health condition, and socioeconomic status.

## CONCLUSIONS

The results of this study indicate that the source and quality of information provided with regard to medication intake during lactation are significantly associated with the initiation, maintenance or cessation of breastfeeding. The implementation of training and consulting services with reflection on the topic of medication intake during lactation should become an integrated part of undergraduate, postgraduate and continuing educational programs in order to be applied efficiently in daily clinical practice by HPs. Advancing HPs’ evidence-based knowledge, communication skills, confidence and competence in breastfeeding management will undoubtedly contribute favorably to breastfeeding indicators on a national and international level.

## Data Availability

The data supporting this research are available from the authors on reasonable request.
